# Editorial: Prevention of viral diseases by gene targeting

**DOI:** 10.3389/fgeed.2024.1395468

**Published:** 2024-03-20

**Authors:** Fan Luo, Qiankun Wang, Shuliang Chen

**Affiliations:** ^1^ State Key Laboratory of Virology and Hubei Province Key Laboratory of Allergy and Immunology, Institute of Medical Virology, TaiKang Medical School, School of Basic Medical Sciences, Wuhan University, Wuhan, China; ^2^ Shenzhen Bay Laboratory, Institute of Infectious Diseases, Shenzhen, China

**Keywords:** infectious diseases, gene therapy, CRISPR/Cas9, genome wide CRISPR screen, host factors

Humans and animals worldwide have been persistently attacked by emerging and re-emerging pathogens. Unfortunately, the majority of these infectious diseases remain incurable due to the lack of effective remedies (Gao, 2018). To counteract the health threat from etiological agents, in addition to the fundamental research regarding pathogenesis and treatment development, it is imperative to globally acknowledge and implement the One Health strategy, the importance of which was convincingly demonstrated during the COVID-19 pandemic (One Health High-Level Expert et al., 2022; One Health High-Level Expert, 2023). Over the past two decades, one of the most inspirational breakthroughs in emerging infectious diseases was the incessant triumph of HIV-1/AIDS “functional cure” via gene therapy with clinical CCR5Δ32/Δ32 stem cell transplantation (Zhang et al., 2022; Hsu et al., 2023; Jensen et al., 2023; Dickter et al., 2024). This achievement sheds light on the successful treatment of infectious diseases by gene targeting. More importantly, with the development and advancement of various gene targeting tools based on CRISPR/Cas9, it is possible to utilize these methodologies to identify novel host genes that regulate viral infections through library screening, elucidate the functions of virally encoded elements through genome modification, and decode the viral lifecycles or infectious states through neo-genetic detection/diagnostics. In this Research Topic, we present two *original research* articles on CRISPR/Cas9 applications in HIV-1 gene editing, along with two *review* papers summarizing the progress of CRISPR screening approaches in infectious diseases, including SARS-CoV-2.

The ability of the CRISPR/Cas9 gene editing system to prevent HIV-1 infection in multi-targeting cells has been verified. However, the efficiency of HIV-1 inhibition mediated by gRNA/Cas9 remains suboptimal due to variable delivery efficiency of gRNA/Cas9 in cells. Allen et al. proposed a two-color system to simultaneously track viral protein and gRNA/Cas9 expression in the same target cell. Utilizing this dual fluorescent system, they standardized the delivery efficiency of gRNA/Cas9 and subsequently evaluated the anti-HIV-1 efficacy of previously designed gRNAs through extensive bioinformatic pipeline analysis (Sullivan et al., 2019). Their findings revealed that gRNAs targeting conserved sites in the 5′portion of the U3 element of LTR are largely ineffective. Whereas gRNAs targeting the TAR region or other conserved sequences of HIV-1 genome demonstrate promising effectiveness, with the selected molecular gRNA target 1 (SMRT1) being the most effective as evidenced by 94.6 percent inhibition of VSV-G pseudo-typed HIV-1 GFP expression. In addition, the combination of gRNAs targeting SMRT1 and TatA further enhances the efficacy of HIV-1 reduction by 96.8 percent. Furthermore, a correlation between predicted gRNA activity, genetic diversity of HIV-1, and observed effectiveness is evident, underscoring the importance of informed gRNA design. These findings highlight the therapeutic potential of CRISPR/Cas9 against HIV-1 and advocate for ongoing research in optimizing gRNA design to achieve maximal therapeutic efficacy while minimizing off-target effects.

The reported strategies for HIV-1/AIDS gene therapy have primarily utilized SpCas9, SaCas9, or CPF1. With the rapid advancement of CRISPR-Cas systems, it is critical to explore the effects of alternative Cas editors on HIV-1 genome editing. Dampier et al. introduced a nominate, diversify, narrow, and filter (NDNF) pipeline that can identify effective Cas enzymes and gRNAs for targeting the HIV-1 genome. Through literature review and computational analysis, they evaluated the influence of various potential Cas editors, mismatch tolerance, PAM recognition sites, and off-target effects on targetable HIV-1 sequences. The results indicate that broader PAM variants increase targetable sites, while tighter mismatch tolerances enhance specificity but limit tolerance to HIV-1 genetic variability. The study also explores the numerical stability of estimates and the efficacy of mutations in the HIV-1 genome. Findings suggest a balance between broad spectrum potential and off-target risks, with a two-mismatch tolerance identified as optimal. Furthermore, modifications to PAM recognition sites influence the number of targetable sites. This research underscores the importance of considering multiple factors when selecting optimal editors for HIV-1 targeting. Overall, the NDNF pipeline offers a comprehensive framework for evaluating Cas enzymes and gRNAs for HIV-1 gene editing, facilitating advancements in the field and guiding future research directions.

The process of pathogen infection and propagation necessitates the involvement of host factors. Understanding the interplay between hosts and pathogens is the foundation for discovery of drug targets and therapeutic strategies to combat microbial infection. Genome-wide CRISPR screens have been approved as a powerful approach to pinpoint key host factors implicated in various stages of viral replication and pathogenesis. Srivastava et al. described the application of genome-wide CRISPR screens in viral diseases caused by the Influenza virus, HIV, Flaviviruses, hepatitis A/B/C virus, Herpesviruses, Sindbis virus, SFTSV, and SARS-CoV-2, as well as bacterial diseases caused by *Staphylococcus aureus*, *Mycobacterium tuberculosis*, *Salmonella sps*, *Legionella pneumophila*, and Enterohemorrhagic *Escherichia coli*. It highlighted the pivotal role of CRISPR screens in comprehending host-pathogen interactions and disease progression by unravelling the involvement of known or novel cellular factors.

Over the past 4 years, the COVID-19 pandemic has presented unprecedented challenges globally, emphasizing the urgent need to uncover the dynamic interplay between SARS-CoV-2 and host cells. Through CRISPR/Cas9-mediated library screening, numerous key host factors crucial for coronavirus infection have been characterized, offering valuable insights into the molecular mechanisms underlying SARS-CoV-2 infection and providing druggable targets for therapy. Cui et al. demonstrated the roles of top-ranked host proteins, such as TMEM41B, TMEM106B, GATA6, and Mucins, in modulating SARS-CoV-2 infection and replication through CRISPR/Cas9-based screening platforms. Furthermore, they delved into the CRISPR/Cas13-based gene therapy strategies of targeting these host factors and emphasized their investigation into the dysfunction of Ctsl to alleviate SARS-CoV-2 infection in mouse models. Despite the challenges encountered, the CRISPR-based approaches hold considerable promise in combating both current and future coronavirus infections, particularly in addressing viral variants and host factors resistant to conventional treatments.

The pandemic presents a long-term threat for human beings and other hosts. To date, we continue to grapple with the challenge of eradicating HIV-1 infection and combating numerous severe viral diseases. However, along with the revolutionary progress of CRISPR/Cas9-based gene targeting tools, remarkable advancements have been made in the field of anti-viral research. These cutting-edge gene editing toolkits and library screens ensure a promising future in the fight against infectious diseases, provided we can surmount challenges in delivery efficiency, off-target effects, and limitations in various organisms.
